# Maternal and Fetal Implications of Oropouche Fever, Espírito Santo State, Brazil, 2024

**DOI:** 10.3201/eid3104.241986

**Published:** 2025-04

**Authors:** João Paulo Cola, Ana Paula Brioschi dos Santos, Raphael Lubiana Zanotti, Adriana Endlich da Silva Dela Costa, Karina Bertazo Del Carro, Lesliane de Amorim Lacerda Coelho, Angelica Espinosa Miranda, Creuza Rachel Vicente

**Affiliations:** Secretaria de Estado da Saúde do Espírito Santo, Vitória, Brazil (J.P. Cola, A.P. Brioschi dos Santos, R. Lubiana Zanotti, A. Endlich da Silva Dela Costa, K. Bertazo Del Carro, L. de Amorim Lacerda Coelho); Universidade Federal do Espírito Santo, Programa de Pós-Graduação em Doenças Infecciosas, Vitória (A.E. Miranda, C.R. Vicente)

**Keywords:** Oropouche virus, viruses, vector-borne infections, pregnancy, congenital abnormalities, infection, transmission, maternal, fetal, Espírito Santo, Brazil

## Abstract

Reemergence of Oropouche fever in Brazil raises concerns about potential risks for infection in pregnancy. We describe a case series of Oropouche fever in pregnant women and their neonates in Espírito Santo State, Brazil, in 2024. Of 73 pregnancies, 15 pregnancies concluded by the end of the study period; of those, 14 resulted in live births and 1 in spontaneous abortion. Placental reverse transcription PCR tests were positive for Oropouche virus RNA in 5 infections in the third trimester. Two infections occurred in the first trimester, resulting in 1 spontaneous abortion and 1 live birth with corpus callosum dysgenesis. Of 13 infections that occurred in the third trimester, 1 showed possible intrapartum transmission with clinical manifestations in the neonate, whereas the others were asymptomatic. We found no anomalies in third-trimester infections. These findings suggest possible vertical transmission of Oropouche virus and a potential link with spontaneous abortion or malformation.

Oropouche fever is a vectorborne viral disease caused by Oropouche virus (OROV), an Orthobunyavirus primarily transmitted through the bite of the *Culicoides paraensis* midge (*1*). Endemic to tropical and subtropical regions of Central and South America, Oropouche fever is a reemerging public health concern with the potential for urban outbreaks and high attack rates (*2*). The disease typically manifests with a febrile syndrome characterized by headaches, myalgias, arthralgias, and occasionally neurologic manifestations, such as meningitis or encephalitis (*3*).

Arboviruses, such as Zika virus, dengue virus, chikungunya virus, yellow fever virus, and emerging and reemerging threats such as OROV, pose substantial risks during pregnancy, potentially leading to severe maternal and fetal outcomes, such as stillbirth (*4*). Although the effects of more established arboviruses are well documented, such as congenital Zika syndrome, increased risk for hemorrhage with dengue, and preterm birth with chikungunya, as well as intrapartum transmission in dengue, chikungunya, and yellow fever (*4*–*10*), the effects of Oropouche fever during pregnancy remain less understood. However, the reports of stillbirth and congenital conditions and the virus’s neurotropic tendencies raise concerns about possible vertical transmission and fetal complications (*6*,*10*,*11*).

In addition to possible vertical transmission and adverse neonatal outcomes, pregnancy’s physiologic and immunologic changes might increase the susceptibility of pregnant women to severe disease and complications (*11*,*12*). Those risks highlight the urgent need for research on Oropouche fever’s implications for maternal and neonatal health, alongside strengthened surveillance, vector control, and preventive measures such as mosquito and midge bite avoidance during pregnancy (*13*).

Oropouche fever is also a substantial concern in areas outside Brazil’s northwestern endemic area because of low population immunity (*1*). During a large outbreak in Espírito Santo, located on the coast of the southeast region of Brazil, we followed a series of cases of Oropouche fever in pregnant women to determine their clinical course and pregnancy outcomes.

## Methods

We conducted a case series study describing the epidemiologic, clinical, laboratory, and obstetric outcomes of pregnant women diagnosed with Oropouche fever and their neonates. The cases included Oropouche fever in the Espírito Santo State’s residents reported during March 28–December 22, 2024. The study is in accordance with the Helsinki Declaration revised in 2013. It has the ethical approval of the Research Ethics Committee of the Health Science Center at the Federal University of Espírito Santo (approval no. 7,004,185). All data were anonymized to protect patient privacy.

We accessed the data through the e-SUS Health Surveillance System (e-SUS VS), the official system for compulsory disease reporting in Espírito Santo. The e-SUS VS system was developed in partnership with the Pan American Health Organization and implemented in January 2020. Laboratory surveillance for OROV in Espírito Santo began on March 25, 2024, after Brazil’s Ministry of Health provided reverse transcription PCR (RT-PCR) reagents to test samples negative for dengue, Zika, and chikungunya viruses at the Central Laboratory of Espírito Santo State (*14*). On April 23, 2024, the State Department of Health reported the first cases of Oropouche fever in the state (*15*). The protocol for managing Oropouche fever during pregnancy includes testing with RT-PCR in the following conditions: all pregnant women with suspected Oropouche fever; a neonate of a pregnant woman with confirmed or suspected Oropouche fever at the time of delivery; or cases of fetal death, stillbirth, and spontaneous abortion in pregnancies in women with confirmed or suspected Oropouche fever. For neonate cases, the test includes the placenta, umbilical cord fluid, and serum from the neonate and the mother, even if the neonate appears healthy. Cerebrospinal fluid is also tested in cases where the neonate shows neurologic complications (*16*).

The case series included pregnant women with a diagnosis of Oropouche fever on the basis of epidemiologic and clinical criteria and laboratory confirmation through RT-PCR. The RT-PCR for OROV was performed on serum samples collected up to 5 days after the onset of symptoms and placenta after delivery, following the protocol of Naveca et al. (*17*). We also included symptomatic pregnant women who were not tested for OROV infection but whose neonates were diagnosed with OROV infection through RT-PCR within 5 days of life. Epidemiologic and clinical criteria included those living in or visiting areas with active OROV transmissions and who had symptoms such as fever, headache, myalgia, back pain, arthralgia, retro-ocular pain, nausea, vomiting, petechiae, and exanthema (*18*). All neonates underwent a physical examination to identify any congenital abnormalities.

We collected data on maternal demographics and clinical characteristics, clinical manifestations of Oropouche fever, laboratory findings, and pregnancy outcomes, including type of delivery and complications such as preterm birth or spontaneous abortion. We also assessed neonatal outcomes, including birthweight and length, Apgar scores, and laboratory evidence of vertical transmission.

We described maternal age as mean + SD, medians and interquartile ranges, and categories divided by decades. Clinical characteristics included underlying conditions (e.g., diabetes and hypertension), week and trimester of illness onset (i.e., first, second, or third), and Oropouche fever clinical manifestations (e.g., fever, headache, myalgia, back pain, arthralgia, retro-ocular pain, nausea, vomiting, petechia, and exanthema). Regarding pregnancy outcomes, the types of delivery were cesarean section or vaginal birth and preterm birth (delivery at <37 weeks). Neonatal outcomes included low birthweight and length, considering the gestational age and Apgar score (reported categorically as 0–6 or 7–10) (*19*). Evidence of vertical transmission included OROV RNA identification by RT-PCR in the neonate biologic samples, such as serum. We summarized the results by absolute and relative frequencies.

## Results

A total of 4,062 cases of Oropouche fever were reported during March 28–December 22, 2024, in Espírito Santo, including in 73 pregnant women and their neonates. Of those, 71 women had OROV infection confirmed by RT-PCR during pregnancy; we included an additional 2 because their neonates had detectable RT-PCR for OROV RNA within 5 days of life.

Most pregnant women were 20–39 years of age (n = 67) and had OROV infection detected in the third trimester of pregnancy (n = 33). Hypertension (n = 6) and diabetes mellitus (n = 1), excluding gestational diabetes, were the only underlying conditions. Assessing whether the hypertension cases were related to chronic underlying hypertension or were pregnancy-induced was not possible. The most reported Oropouche fever manifestation was headache (n = 59), followed by fever (n = 55), myalgia (n = 52), retro-ocular pain (n = 32), nausea (n = 29), and back pain (n = 28) (Table 1).

**Table 1:** Demographic and clinical characteristics of 73 pregnant women with Oropouche virus infection in Espírito Santo State, Brazil, March 28–December 22, 2024*

Characteristic	Pregnancy ongoing, n = 58	Pregnancy concluded, n = 15	Total
Age, y			
Median (IQR)	29 (24–34)	28 (25–31)	29 (24–33)
Mean (+SD)	28.70 (5.73)	28.73 (4.84)	28.71 (5.52)
18–19	4 (6.90)	0	4 (5.48)
20–29	26 (44.83)	9 (60.00)	35 (47.95)
30–39	27 (46.55)	5 (33.33)	32 (43.84)
40–42	1 (1.72)	1 (6.67)	2 (2.74)
Underlying condition			
Hypertension	6 (10.34)	NA	6 (8.22)
Diabetes	1 (1.72)	NA	1 (1.37)
Pregnancy trimester during Oropouche fever			
First	12 (20.69)	2 (13.33)	14 (19.18)
Second	26 (44.83)	NA	26 (35.62)
Third	20 (34.48)	13 (86.67)	33 (45.21)
Oropouche fever clinical manifestation			
Fever	42 (72.41)	13 (86.67)	55 (75.34)
Headache	46 (79.31)	13 (86.67)	59 (80.82)
Myalgia	41 (70.69)	11 (73.33)	52 (71.23)
Back pain	23 (39.66)	5 (33.33)	28 (38.36)
Arthralgia	8 (13.79)	2 (13.33)	10 (13.70)
Retro-ocular pain	27 (46.55)	5 (33.33)	32 (43.84)
Nausea	23 (39.66)	6 (40.00)	29 (39.73)
Vomiting	11 (18.97)	NA	11 (15.07)
Petechia	4 (6.90)	1 (6.67)	5 (6.85)
Exanthema	9 (15.52)	2 (13.33)	11 (15.07)

*Values are no. (%) except as indicated. IQR, interquartile range; NA, not applicable.

Of the 73 pregnancies under observation, 15 had concluded by the end of the study period; 14 resulted in live births, and 1 resulted in spontaneous abortion. The remaining 58 pregnancies were ongoing, with patients continuing to receive monitoring. At the conclusion of this data collection period, no fetal anomalies had been reported in those cases.

In 1 case of spontaneous abortion (case 1), a pregnant woman without underlying conditions reported fever, headache, myalgia, back pain, retro-ocular pain, nausea, and arthralgia at 7 weeks of gestation; her serum sample tested positive for OROV RNA by RT-PCR. She sought hospital care at 8 weeks of pregnancy after experiencing vaginal bleeding. Imaging tests confirmed the spontaneous abortion (Table 2).

**Table 2:** Description of Oropouche virus infections in 15 concluded pregnancies, Espírito Santo State, Brazil, March 28–December 22, 2024*

Case	Clinical manifestations in pregnant woman	Weeks of gestation at diagnosis	RT-PCR of pregnant woman	RT-PCR of placenta	Clinical manifestations in neonates	RT-PCR in neonate	Outcomes
1	Fever, headache, myalgia, back pain, retro-ocular pain, nausea, and arthralgia	7	Positive	NA	NA	NA	Spontaneous abortion at 8 weeks of pregnancy
2	Fever, myalgia, headache, nausea, retro-ocular pain	7	NA	NA	Asymptomatic	Positive	Dysgenesis of the corpus callosum
3	Fever, headache, myalgia	33	Positive	Negative	Asymptomatic	Negative	No alterations
4	Rash	33	Positive	Negative	Asymptomatic	Negative	No alterations
5	Headache, myalgia	33	Positive	Negative	Asymptomatic	NA	No alterations
6	Fever, headache, myalgia, nausea	34	Positive	Positive	Asymptomatic	Negative	No alterations
7	Fever, headache, myalgia, nausea	35	Positive	Positive	Asymptomatic	Negative	No alterations
8	Fever, headache, myalgia, back pain	37	Positive	Positive	Asymptomatic	Negative	No alterations
9	Fever, headache, myalgia, back pain and rash	37	Positive	Negative	Asymptomatic	NA	No alterations
10	Fever, headache, myalgia	38	NA	NA	Fever, exanthema, and agitation at 4 d of age	Positive	Possible intrapartum transmission
11	Fever, headache, myalgia, and arthralgia	38	Positive	Negative	Asymptomatic	Negative	Hyperthermia at birth
12	Fever, headache, myalgia, back pain	39	Positive	Positive	Asymptomatic	Negative	No alterations
13	Fever, headache, myalgia, retro-ocular pain, nausea,	40	Positive	Positive	Asymptomatic	Negative	No alterations
14	Fever, headache, myalgia, back pain and arthralgia	40	Positive	Negative	Asymptomatic	NA	No alterations
15	Fever, headache, myalgia, retro-ocular pain, nausea	40	Positive	Negative	Asymptomatic	Negative	No alterations

*Physical examinations were performed in all cases. Data on brain imaging was not available except for case 2. NA, not applicable, RT-PCR, reverse transcription PCR.

Among the 14 deliveries performed, most were cesarean sections (n = 10), with gestational ages >37 weeks (n = 14). The weight, length, and Apgar scores were adequate in all neonates (Table 3).

**Table 3:** Pregnancy and neonatal outcomes in 14 pregnant women with Oropouche virus infection and concluded pregnancy, Espírito Santo State, Brazil, March 28–December 22, 2024*

Characteristic	No. (%)
Type of birth	
Cesarean	10 (71.43)
Vaginal	4 (28.57)
Gestational age at birth	
Term, 37–41 wks	14 (100.0)
Birthweight and length	
Adequate	14 (100.00)
Apgar score >7	14 (100.00)
Positive RT-PCR for Oropouche virus RNA	
Placenta	5 (38.46)
Neonate	2 (15.38)

*Excludes 1 case (1.37%) of spontaneous abortion. No cases were preterm (<37 weeks), had low birthweight and length, had an Apgar score of 0–6, or had detectable Oropouche virus RNA in umbilical cord fluid. RT-PCR, reverse transcription PCR.

One pregnant woman (case 2) without underlying conditions had fever, headache, myalgia, nausea, and retro-ocular pain at 7 weeks of pregnancy and did not have laboratory confirmation of diagnosis for OROV or other arboviruses. At 32 weeks of gestation, ultrasound findings suggested dysgenesis of the corpus callosum body (truncus) on the right with dilation of the body of the lateral ventricle. At birth, through cesarean section at 40 weeks of gestation, cranial ultrasound findings confirmed dysgenesis of the corpus callosum. No other abnormalities were noted during the physical examination, and the neonate survived. The RT-PCR was positive for OROV RNA in the neonate’s serum sample 1 day after birth. No additional data on other imaging tests or ophthalmologic evaluations were available (Table 2).

Five of the pregnant women who underwent delivery had positive RT-PCR for OROV RNA in the placenta fragment, all of them with OROV infection in the third trimester of pregnancy. In 2 cases (cases 2 and 10), evidence of vertical transmission was found, and RT-PCR confirmed OROV RNA in the serum samples of the neonates (Table 2).

Possible intrapartum transmission during cesarean section was observed (case 10). In this case, spontaneous rupture of the membranes occurred 2 hours before the surgical delivery. A neonate returned to the hospital at 4 days of age, 1 day after the postpartum discharge, with fever; maculopapular rash in the torso, legs, and arms; and agitation. RT-PCR confirmed OROV RNA in a serum sample but was negative for dengue, Zika, chikungunya, Mayaro, and West Nile viruses RNA. The neonate was discharged after 6 days of hospitalization and was healthy at 10 days of life. The mother had no underlying conditions and reported fever, headache, and myalgia 5 days before delivery. Laboratory tests for OROV were not performed. Intra-household contacts had OROV RNA detected by RT-PCR in serum samples in the same period (Table 2).

Therefore, among the 2 infections reported in the first trimester of pregnancy, 1 resulted in spontaneous abortion and the other in a live-born infant with dysgenesis of the corpus callosum but no other apparent anomalies. Of the 13 infections reported in the third trimester, 1 possible intrapartum transmission occurred (i.e., a neonate had clinical manifestations) and 11 were asymptomatic cases. We found no anomalies in pregnancies affected by third-trimester infections (Table 2).

## Discussion

This case series highlights the potential implications of OROV infection during pregnancy, including evidence of peripartum transmission supported by a neonate with a positive test manifesting Oropouche fever–related signs and symptoms. In addition, a case of spontaneous abortion and a case of fetal dysgenesis of the corpus callosum were reported. The detection of OROV RNA in the placenta was not related to its detection in the serum samples of neonates, and those cases did not have congenital abnormalities.

Only recently, with the spread of the disease to extra-Amazon regions of Brazil and the increasing transmission in the human population, have severe cases of Oropouche fever started to be reported, including deaths, spontaneous abortions, stillbirths, and congenital conditions such as microcephaly (*20*,*21*). The emergence of a novel OROV reassortant lineage might be linked with those cases, as suggested by previous studies (*4*,*22*–*24*).

This case series from Espírito Santo suggests a potential link between first-trimester OROV infections and adverse outcomes, including 1 spontaneous abortion and a live-born infant with a brain abnormality, despite spontaneous aborting being a common occurrence in pregnancy. In contrast, 13 third-trimester infections resulted in healthy term deliveries. However, further research is needed to confirm this association, given that adverse pregnancy outcomes have also been reported in late pregnancy infections (*4*). Although third-trimester infections resulted in live births, 5 cases showed evidence of placenta infection, and 1 involved possible intrapartum transmission after spontaneous rupture of the membranes followed by cesarean, resulting in neonatal disease. Cases of intrapartum transmission are also reported in cases of dengue, chikungunya, and yellow fever infections (*5*). In this case, the period between the postpartum discharge and the neonate hospital admission with Oropouche fever clinical manifestations was 1 day, reinforcing the improbability of infection by vector biting and the possibility of intrapartum transmission.

Considering the number of pregnant women with confirmed OROV infection at different stages of pregnancy under follow-up in Espírito Santo, the findings among those who delivered highlight the possibility of increasing unfavorable outcomes. Therefore, ongoing evaluation of the effects of infection at different stages of pregnancy and its outcomes is necessary (*25*). Of note, no instances of preterm birth, low weight and length, or an Apgar score <7 were reported. The association between preterm birth and OROV infection remains unclear (*21*).

The first indication of possible OROV vertical transmission in Brazil was reported by the Ministry of Health in July 2024 on the basis of identification of OROV-specific antibodies in serum and cerebrospinal fluid samples from 4 neonates with microcephaly and in 1 case of fetal death in which OROV was detected in the umbilical cord, placenta, and various organs (*26*). Spontaneous abortions, stillbirths, and congenital conditions linked to OROV infection have been reported in Pernambuco, Manaus, Acre, Ceará, Bahia, and Pará States (*4*,*21*,*27*,*28*). Similar outcomes after vertical transmission have been observed in other Orthobunyavirus-infected pregnant animals and humans (*29*).

Despite the impossibility of establishing causality about the infection’s teratogenic effects in those studies, experimental findings in neonatal mice submitted to subcutaneous inoculation with OROV showed infection affecting the central nervous system, especially the posterior parts of the brain, reaching the spinal cord and spreading to the brain parenchyma (*30*). In addition, mice neurons were the target cells affected by OROV, having glial reaction, astrocyte activation, and neuronal apoptosis (*31*). The corpus callosum dysgenesis reported in Espírito Santo and Acre States support these findings (*27*). In adults, neurologic disease and the presence of OROV in cerebrospinal fluids were previously reported (*32*,*33*), and in vitro experiments demonstrated an inflammatory response and tissue damage when human neural cells were infected with OROV (*34*). In addition, an analogy of Zika virus infection leading to congenital Zika syndrome is plausible, because both Zika virus and OROV manifest in neurotropism and can cross the placental barrier (*35*). Those findings contribute to support the biological plausibility of teratogenic effects resulting from OROV infections. Nevertheless, prospective studies are needed to confirm causality by assessing temporality, association strength, and consistency (*36*,*37*). Such studies should also approach complex models of multicausality that consider social and other determinants. The mechanisms of placental transmission, maternal immunity, and fetal susceptibility also require further investigation (*27*).

Espírito Santo, as the state with the highest incidence of Oropouche fever cases outside the original endemic region in Brazil, underscores the effect of epidemics in areas with high vectorial presence and an immune-naive population, including a considerable incidence among women of reproductive age (*18*). This scenario raises concerns about pregnant women visiting those areas, particularly after the US Centers for Disease Control and Prevention issued recent advice to avoid nonessential travel to Espírito Santo because of the current local epidemiologic situation, marked by high incidence and reports of suspected and confirmed deaths (*38*,*39*). Transmission in the state primarily occurs in small cities, where ecologic conditions, such as plantations providing organic matter and humidity, are ideal for breeding *C. paraensis* mosquitoes, given that those conditions support the laying of eggs by the female (*22*). Therefore, this experience serves as a warning for other areas in Brazil to which Oropouche fever is not endemic. Health professionals in such areas must be vigilant, ensuring that illnesses during pregnancy are further investigated to avoid complications. Of note, this case series includes reports of pregnant women with OROV infections in the absence of fever, which must be considered by clinicians and for surveillance purposes. The Oropouche fever outbreak emphasizes the need for surveillance systems to adapt quickly to emerging and reemerging infectious disease threats.

Our study also highlights the need for effective protocols for preventing and managing OROV infection in pregnant women. The previous experience with congenital Zika syndrome could contribute to defining these actions. Suggested measures included considering Oropouche fever as a differential diagnosis for febrile illness, providing laboratory tests for symptomatic pregnant women aiming for early diagnosis, conducting serial ultrasounds to monitor fetal malformations and growth restriction, performing developmental and neurologic evaluations of neonates, and counseling pregnant women on vector biting protection, sexual protection, and avoiding travel to endemic areas (*28*,*38*,*40*–*42*). RT-PCR should be performed within 5 days of symptom onset, and pregnant women should be encouraged to seek medical care promptly. The test used in Espírito Santo has an amplification efficiency >98% and a limit of detection ranging from 2 to 20 copies/reaction, but it has not yet been compared with other diagnostic tests, and the possibility of false-positives or false-negatives must be considered (*17*). The incubation period for OROV infection ranges from 3 to 10 days (*41*).

Moreover, the response in endemic regions should focus on establishing robust monitoring systems to detect and report cases early, genomic surveillance, educating communities on preventing vector exposure, and ensuring health services and healthcare professionals are equipped to recognize and manage Oropouche fever cases, including congenital and neonatal disease cases. Future studies should investigate the viral, vector, human, and environmental determinants of OROV spread and outcomes, including its urbanization, by using the One Health approach (*41*). This comprehensive perspective will help prevent and manage cases during pregnancy and in neonates.

In conclusion, Oropouche fever in pregnancy might result in vertical (including intrapartum) transmission, potentially leading to spontaneous abortion and fetus malformation. Further investigations are necessary to establish causality between infection during pregnancy and these outcomes. Meanwhile, health systems, healthcare professionals, and communities must be prepared to prevent, detect, monitor, and respond to OROV infection during pregnancy and provide appropriate follow-up and treatment to the mothers and neonates affected.
